# Clinical Advantages and Limitations of Monolithic Zirconia Restorations Full Arch Implant Supported Reconstruction: Case Series

**DOI:** 10.1155/2015/392496

**Published:** 2015-06-01

**Authors:** Joao Carames, Loana Tovar Suinaga, Yung Cheng Paul Yu, Alejandro Pérez, Mary Kang

**Affiliations:** ^1^Faculty of Dental Medicine at the University of Lisbon, Lisbon, Portugal; ^2^Ashman Department of Periodontology and Implant Dentistry, New York University College of Dentistry, 345 East 24th Street, Suite 3W, New York, NY 10010, USA

## Abstract

*Purpose*. The purpose of this retrospective case series is to evaluate the clinical advantages and limitations of monolithic zirconia restorations for full arch implant supported restorations and report the rate of complications up to 2 years after insertion. *Materials and Methods*. Fourteen patients received implant placement for monolithic zirconia full arch reconstructions. Four implants were placed in seven arches, eleven arches received six implants, two arches received seven implants, two arches received eight implants, and one arch received nine implants. *Results*. No implant failures or complications were reported for an implant survival rate of 100% with follow-up ranging from 3 to 24 months. *Conclusions*. Monolithic zirconia CAD-/CAM-milled framework restorations are a treatment option for full arch restorations over implants, showing a 96% success rate in the present study. Some of the benefits are accuracy, reduced veneering porcelain, and minimal occlusal adjustments. The outcome of the present study showed high success in function, aesthetics, phonetics, and high patient satisfaction.

## 1. Introduction

Full arch implant supported restorations have been documented to have high success rates [1–6]. Many combinations of materials have been used for this type of restorations such as metal alloy-acrylic, metal alloy-composite, and metal alloy-ceramic [1, 5, 7]. However, complications including fractured or debonded acrylic resin teeth, wear of opposing surfaces, ceramic chipping, difficulty in shade matching of acrylic and pink ceramic, lack of passive fit, and extensive work for repair after framework breakage have encouraged dentists to look for other material options [1, 5, 7]. The use of zirconia for frameworks is an option that has been proposed [2, 7, 8].

Zirconium oxide is a material that has shown increased popularity in contemporary dentistry [3, 9]. Many studies have shown excellent physical, mechanical, biological, and chemical properties of this material [3, 5, 9, 10]. Fixed dental prostheses were designed and milled in a one-piece zirconia substructure and veneering porcelain was then directly fired onto the substructure [3, 4]. Nevertheless, some reports have documented veneering ceramic fractures (chipping) [1–12] and fractures of the zirconia substructure [1, 3–5].

To overcome these problems, CAD/CAM one-block milled monolithic zirconia was introduced as an alternative for the treatment of implant supported full arch reconstructions [3–5, 12]. The fabrication of the structure in one block reduces breakage possibilities and avoids chipping [4, 5]. Moreover, high strength, minimal occlusal adjustment, and accuracy are some of its advantages [3, 4, 6, 13].

Short-term available data indicates that full contour zirconia framework can be used successfully in implant dentistry [3, 7]. Seven articles involving full contour zirconia restorations have been published (Table 1). Five articles were case reports [1, 5, 9, 12, 13]. One retrospective study with 3- to 5-year follow-up was published in 2013 [10], and one prospective study with 3-year follow up was published in 2010 [11]. Further research is required to evaluate the long-term outcome of monolithic zirconia restorations. Studies of material inherent accelerated aging [3] and wear of opposing dentition are necessary [5].

The purpose of this retrospective case series was to evaluate the clinical advantages and limitations of monolithic zirconia restorations for full arch implant supported restorations and report the rate of complications up to 2 years after insertion.

## 2. Materials and Methods

Clinical data in this study was obtained from the implant database (ID) in the Ashman Department of Periodontology and Implant Dentistry at New York University College of Dentistry. This dataset was extracted as deidentified information from the routine treatment of patients in the department. The ID was certified by the Office of Quality Assurance at NYUCD. This study is in compliance with the health insurance portability and accountability act (HIPAA).

### 2.1. Study Subjects

Patients which referred to New York University Ashman Department of Periodontology and Implant Dentistry in need of prosthetic full arch fixed reconstruction in maxilla, mandible, or both were consecutively selected. The inclusion criteria included patients at least 21 years old, with edentulous maxilla and/or mandible and at least four to nine implants needed to be placed and osseointegrated. Fourteen patients (Four females and ten males with a mean age of 56 years old, range: 37–67) met the inclusion criteria. Each subject selected for this study from the ID had undergone the fabrication of monolithic zirconia frameworks for full arch implant supported reconstructions. Twelve of these patients required maxillary and mandibular full arch reconstruction, and two involved only the maxillary arch. In the two maxillary reconstructions the opposing dentition was in one patient natural teeth and a fixed prosthesis and in the other a complete mandibular denture. A total of twenty-six edentulous arches were restored: fourteen maxillary and twelve mandibular arches. Patients were informed about the prosthetic protocol, risks, and alternatives of treatment.

All complications after delivery were recorded at each follow-up visit up to 3 years. Failures were defined as any defect in the restorations that required the fabrication of a new restoration such as fracture and misfitting. Complications were defined as any defect in the restorations that required repair by laboratory technicians or correction of clinicians such as chipping of veneers (lab) and screw loosening (clinician).

### 2.2. Procedures

**Figure 1:**
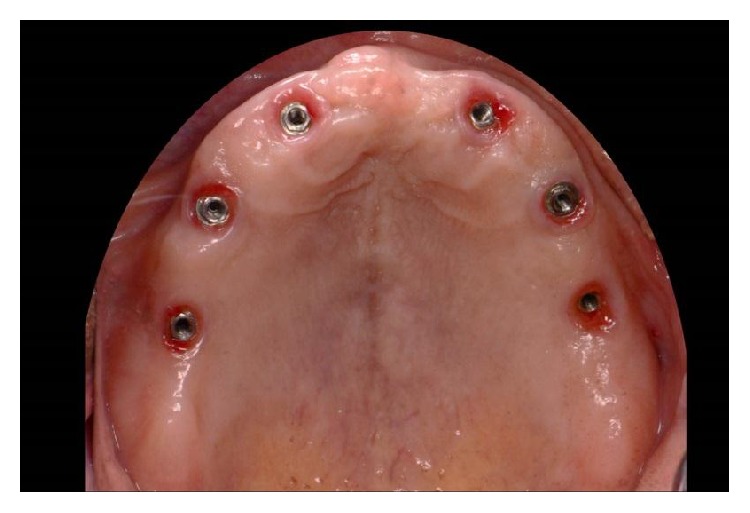
Maxillary occlusal view after tissue healing.

**Figure 2:**
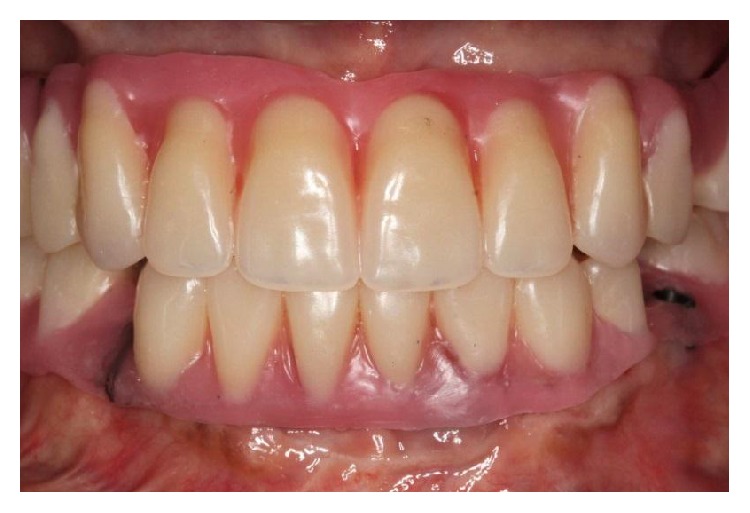
Intraoral frontal view of artificial teeth arrangement.

**Figure 3:**
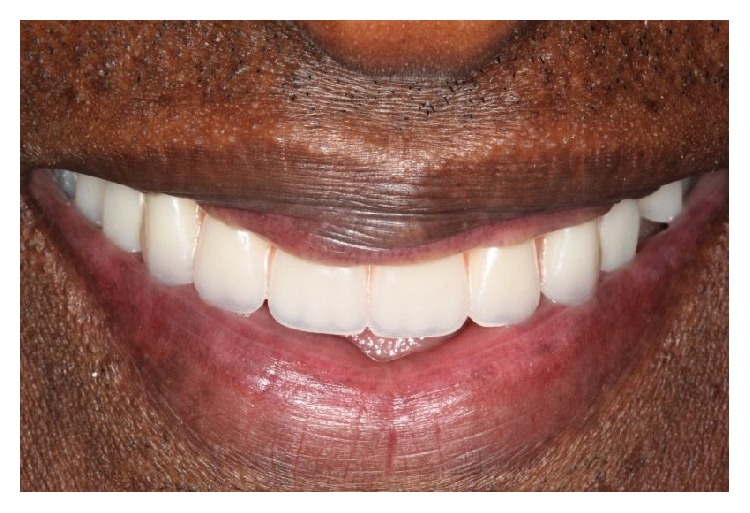
Smile view with artificial teeth arrangement.

**Figure 4:**
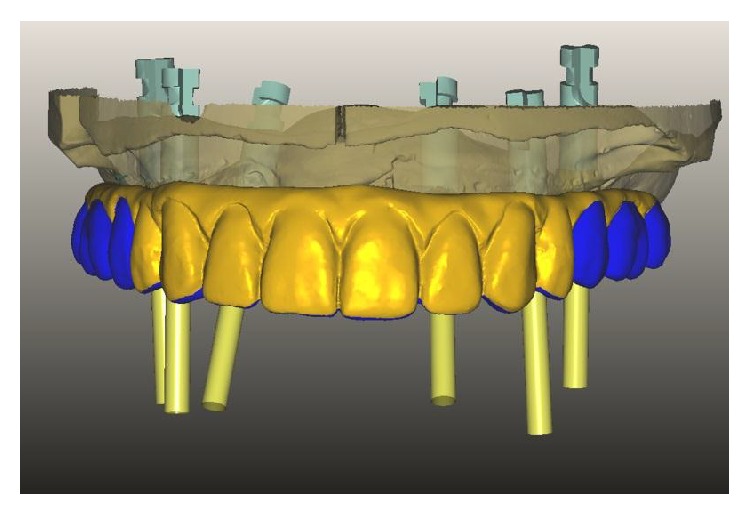
Digital preview of the maxillary monolithic prosthesis.

**Figure 5:**
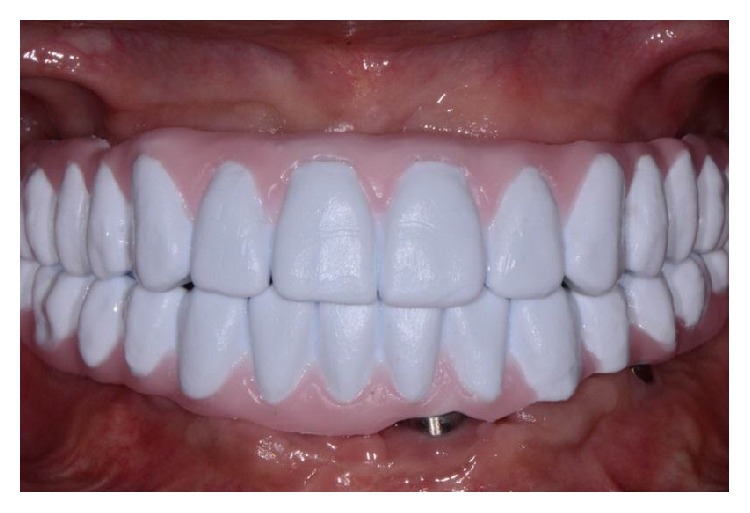
Intraoral frontal view with the epoxy resin prototype.

**Figure 6:**
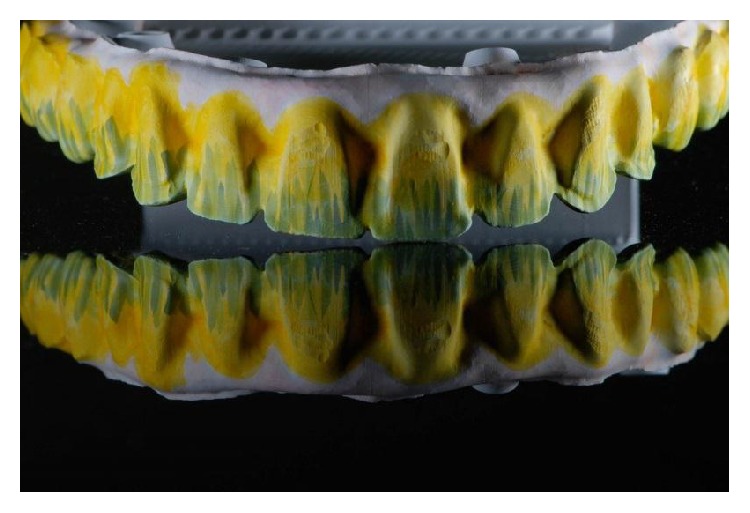
Maxillary monolithic prosthesis with teeth characterization.

**Figure 7:**
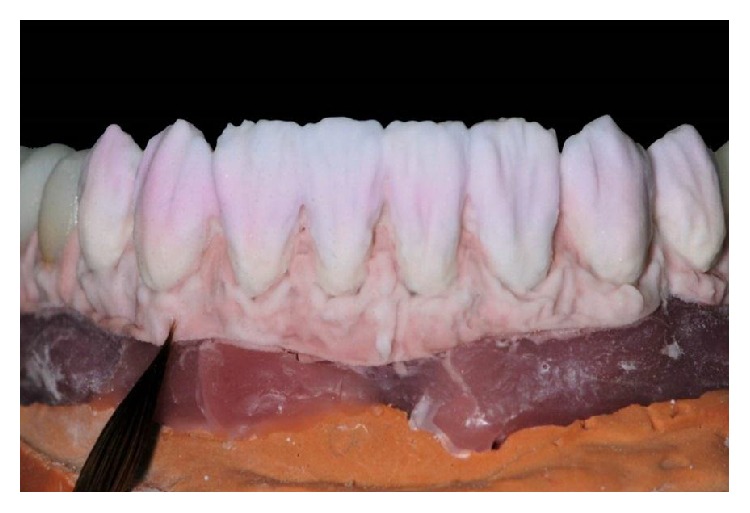
Ceramic application for gingiva colors and teeth ceramic.

**Figure 8:**
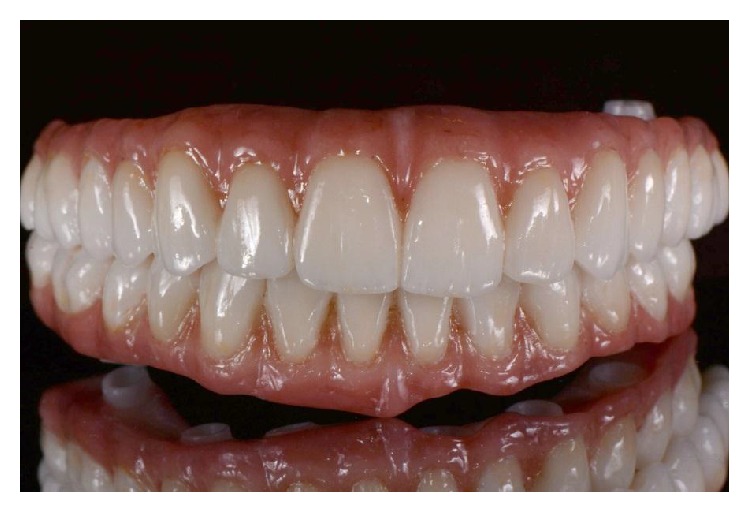
Prostheses after final sintering.

**Figure 9:**
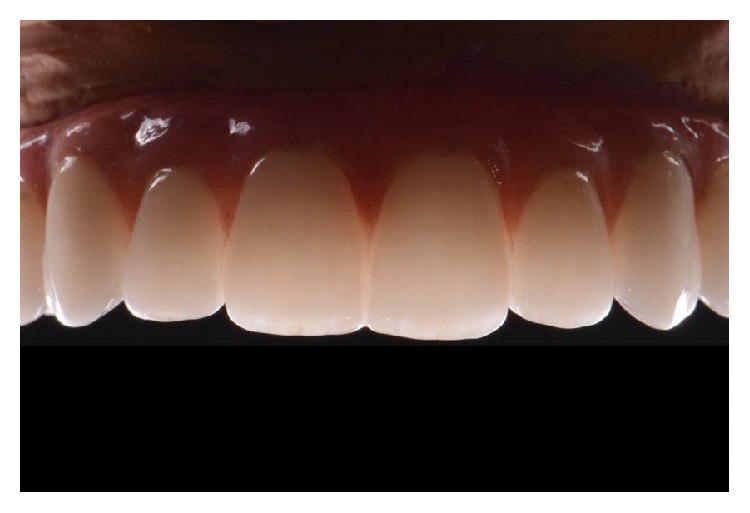
Translucency effect in the anterior maxilla after application of ceramics in the digital cut back.

**Figure 10:**
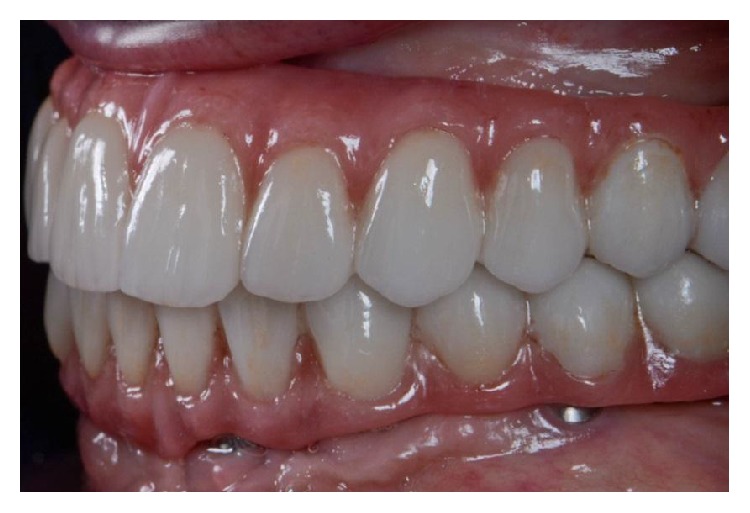
Intraoral lateral view of the final prostheses.

**Figure 11:**
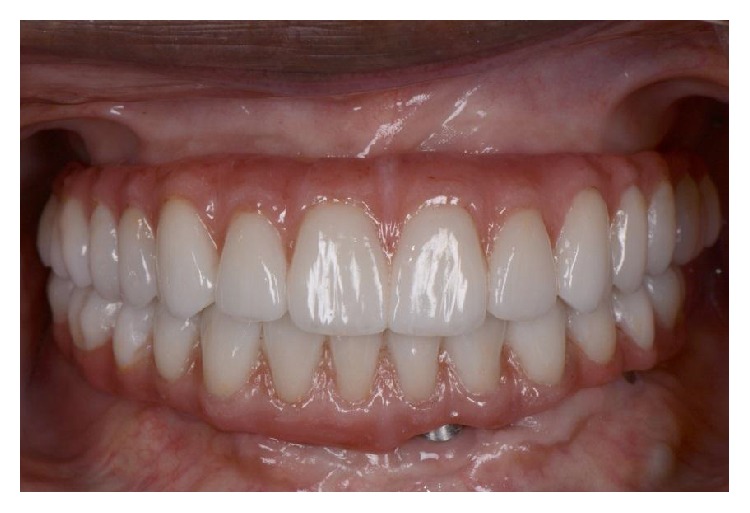
Intraoral frontal view of the final prostheses.

**Figure 12:**
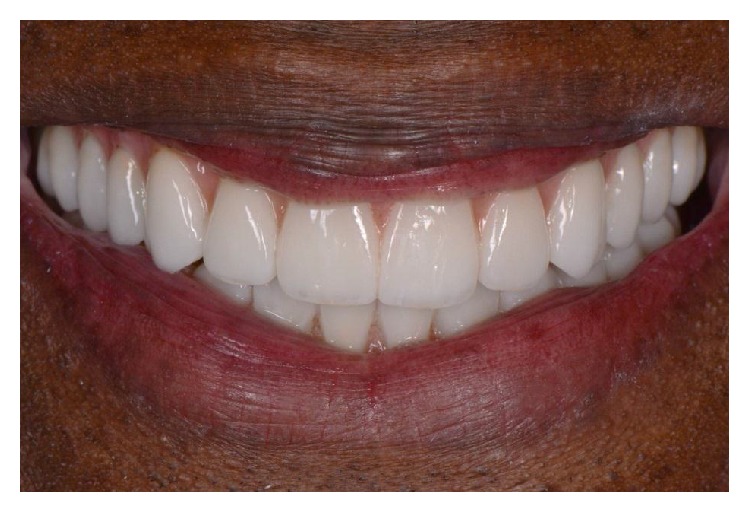
Smile view of the final prostheses.

1. Diagnostic alginate impressions (Jeltrate Plus, Denstply, Milford, DE, USA) were made and poured with model stone (Microstone ISO type 3, WhipMix, Louisville, KY, USA). Occlusal rims were fabricated and adjusted intraorally. Interocclusal records, face bow registration, and centric relation records were taken. Casts were articulated and artificial teeth arrangements (ATA) were performed. Additionally, ATA were duplicated to obtain radiographic and surgical guides. Patients were sent for Cone Beam Computed Tomography (CBCT) scans to evaluate bone dimension and implant positioning (Figures 2 and 3).
2. Four to nine dental implants were placed in the edentulous arches using surgical guides. A one-stage surgical procedure was performed according to the implant planning. The surgical protocol followed the manufacturer's instructions. External connection implants were placed in twenty-three arches, and internal connection implants with intermediate abutments were placed in three arches. The healing time prior to the prosthetic phase was 12 weeks. (Figure 1).
3. During the healing period and until the prosthetic phase was completed, patients wore transitional complete dentures.
4. Fixture level impressions were made of polyether impression material (Impregum, 3M ESPE, St. Paul, MN, USA) in custom light cure resin trays (TRIAD Blue TruTray Visible Light Cure, Dentsply, York, PA, USA). The master cast was made of a reproduction of the gingival soft tissue using a polyvinylsiloxane, addition-type silicone (GI-Mask, Coltene/Whaledent, Cuyahoga Falls, OH, USA) and resin fortified, low expansion die stone (ResinRock ISO Type 4, WhipMix, Louisville, KY, USA). In two patients, in which opposing arches were not restored as full arch reconstructions, alginate impressions were made (Jeltrate Plus, Dentsply, Milford, DE, USA) with stock disposable perforated trays (COE Spacer trays, GC America Inc, Chicago, IL, USA). Interocclusal registrations were made with wax rims (TRIAD Pink Denture Base Regular Pink Fibered, York, PA, USA and Pink Wax Bite Blocks, Keystone, Cherry Hill, NJ, USA). Face bow registration and centric relation record were taken. Artificial teeth arrangements with acrylic dentures teeth (Portrait IPN Dentsply, Milford, DE, USA) were placed in an adequate position to achieve esthetics, phonetics, and vertical dimension in occlusion. Bite registration was taken with vinyl polysiloxane material (BLU Bite HP, Henry Schein, Melville, NY, USA).
5. The laboratory procedures were performed according to the manufacturer's instructions (Zirconia Prettau, Zirkonzahn, Neuler, Germany) at an authorized laboratory. The master cast, opposing cast articulated, and artificial teeth arrangement were scanned to determine the interocclusal relationship to the software (Zirkonzahn.software, Zirkonzahn, Neuler, Germany). According to the corresponding implant type, connectors are milled in titanium to fit in the master cast and scanned again. The prostheses were designed on the software. An epoxy resin prototype was milled and sent for try-in to ensure adequate fit, function, esthetics, and phonetics. After some minor adjustments, the restoration was milled in a monolithic zirconia block. Sixteen of the 26 full arch restorations were digitally cut back on the anterior area to improve esthetics. Characterizations of teeth were made in the monolithic framework (Colour Liquid Prettau, Zirkonzahn, Neuler, Germany). The final restorations were sintered in the oven (Keromikofen 1500, Zirkonzahn, Neuler, Germany). Framework fitting was verified in the master cast. Soft tissue ceramic (ICE Zirkon Keramik Tissue Shades, Zirkonzahn, Neuler, Germany) and ceramic (ICE Zirkon Ceramics and Stains Prettau, Zirkonzahn, Neuler, Germany) according to the shade selection was applied on the framework for esthetic results. Additionally, the prosthesis was sintered overnight. Last working step was placement and bonding of the titanium sleeves into the milled zirconia framework. Finally, prostheses were glaze-fired and sent for delivery. (Figures 4, 5, 6, 7, 8, and 9).
6. The final full arch prostheses were clinically verified with one screw test for passive fit. Moreover, periapical radiographs were taken for radiographic examination. All patients approved and agreed with shape and shade of finals restorations. (Figures 10, 11, and 12).
7. Occlusal screws were torqued following manufacturer's instructions. Gutta-percha was placed in all access holes. In screw-retained restorations, a light-cure microhybrid composite (Z100 Restorative, 3M, St Paul, MN, USA) with proper shade was used to close the access hole. In screw-/cement-retained, fixed prostheses were cemented with temporary cement (TempBond, Kerr, Orange, CA, USA) and excesses were cleaned. The importance of removing the temporary cement and recementing every year, to avoid cement wear and loosening of the prostheses, was explained to the patients.
8. On the day of delivery, alginate impressions were made to fabricate full-cover maxillary night guards. A week later, patients received the night guards and were instructed to wear them at night.
9. Recall appointments were performed after 2 weeks and 3 months after insertion. A yearly appointment is required for clinical and radiographic examination.

## 3. Results

Fourteen patients received implant placement for monolithic zirconia full arch reconstructions. Four implants were placed in seven arches, eleven arches received six implants, two arches received seven implants, two arches received eight implants, and one arch received nine implants. No implant failures or complications were reported for an implant survival rate of 100% with follow-up ranging from 3 to 24 months (Table 2).

Previous restorations of the total arches were as follows: sixteen had nonrestorable teeth or previously failed fixed prostheses, five arches with teeth and removable partial dentures, three complete dentures, and two metal alloy-acrylic prostheses.

Of the twenty-six full arches, twenty-four were implant supported screw-retained, and two full arches were combined implant supported screw-/cement-retained (Table 2). Seventeen full arch restorations were digitally cut back on the buccal surface of the anterior area to improve esthetics. Nine full arch restorations were designed without veneering porcelain. All prostheses were in function at the time of the follow-up, which was from 3 months up to 3 years and 6 months, whereas fourteen arches were followed up up to 1 year. All monolithic zirconia prostheses were clinically and radiographically examined. No defects of the prosthesis were detected and no frameworks needed to be remade. However, a chip-off fracture of the ceramic veneer occurred in 1 of 26 restorations (Table 2, case #8), giving a prosthetic success rate of 96%. The only chip-off fracture occurred in the buccal surface of the veneer in a left central incisor after one year of insertion. A ceramic laminate was used to restore the chip-off. No fracture of the monolithic zirconia frameworks or any other mechanical complications such as screw loosening or decementation of the prostheses were reported. No patient complaints regarding their prosthesis esthetic or function were record.

## 4. Discussion

Improved clinical performance can be expected to be achieved by using monolithic zirconia restorations [2, 10, 11]. Clinical studies have shown increased values of strength and toughness for monolithic zirconia compared to zirconia frameworks with laminate veneering [3, 5]. It has also been shown to result in high standards of esthetics and a reduced amount of metal used in the oral cavity [5, 10]. Full arch monolithic zirconia restorations have shown similar overall survival when compared with high-nobel alloy-based metal ceramic restorations [14]. No bulk fractures or failures in the framework had been reported in the literature with a follow-up of 8 years [2]. The result of the present retrospective case series is in accordance with these trends as no flaws in the monolithic framework occurred during the follow-up examinations.

Several different complications have been related to the use of hybrid prostheses with implants, such as fractures of titanium framework and gold alloys over 5 years [15] and fracture or wear of acrylic teeth due to poor bonding of acrylic to the framework [16]. With ceramometal restorations, chipping or fracture of the ceramic is due to different factors. These include impact and fatigue load, occlusal forces, differences in thermal expansion coefficients, low elastic modulus of the metal, improper design, microdefects, and trauma. Extensive work for repair is required after framework failures [1]. A full monolithic zirconia occlusal contour appears to be a solution to this complication [5, 9, 11, 12]. The present study supports these findings, as twenty-five of 26 monolithic zirconia restorations presented no complications during the follow-up period.

However, chipping of veneering ceramic is a frequent complication of zirconia-based restorations on teeth and implants [3, 4, 7, 9, 11] and sometimes cannot be solved by ceramic polishing [10]. The exact reason for veneer chipping in zirconia core restorations is unclear. Three factors generally play an important role such as interfacial bonding, match of the core-veneer materials, and strength of the veneering ceramic. Also the veneering technique has a potential effect on the chipping of the ceramic due to the processing methods of ceramic, which include repeated sintering in the oven [4]. To overcome chipping fractures of veneered zirconia restorations, laboratory technicians and clinicians should follow precise steps in manufacturing zirconia-based restorations with the knowledge that zirconia as a framework material is highly susceptible to surface modifications and improper laboratory and clinical handling technique [3]. Chip-off fractures of the veneering ceramics have been associated with roughness of the veneering ceramic because of grinding or occlusal function [3, 11]. Analysis of crack propagation direction showed that the chipping failure had originated from roughness of the ceramic at the occlusal region of the cusps. Occlusal adjustments should only be performed with fine grain diamonds, followed by thorough polishing sequence [3]. The use of digital cut back in the monolithic zirconia prevents roughness on the surface that produces the crack propagation and chipping of the veneering. In reference to the present study, of the seventeen arches that were digitally cut back in the anterior area, only one veneer chipping was recorded at 1-year follow-up.

Translucency has been considered one of the primary factors in controlling the esthetic outcome of ceramic restorations. Zirconia has been considered an opaque material compared to other dental ceramic. A recent report has shown some degree of translucency in the zirconia, which was less sensitive to thickness compared to lithium disilicate and leucite-free porcelain. However, translucency of the zirconia ceramics also increased exponentially as the thickness decreased [17]. The digital cut back in the monolithic zirconia allows the restorations to have some degree of translucency. One of the problems with glass ceramics for monolithic restorations is that due to low flexural strength values (360–400 MPa for lithium disilicate) frameworks are prone to fracture when subjected to occlusal loads. Moreover, the use of zirconia frameworks with glass ceramic veneers has been described to have high rates of chipping. In our study zirconia veneers in monolithic restorations resulted in high esthetic, good mechanical properties, and less complications.

Wear rates of the enamel opposing zirconia ceramic have been reported, showing cracks or even fractures in all the ridges. The hardness and thickness of enamel, chewing behavior, parafunctional habits, and neuromuscular forces as well as abrasive nature of food can influence clinical wear. Due to the elasticity modulus of 210 GPa of zirconia and hardness of 1200 Vickers Hardness, some enamel wear is expected. Moreover, some reports have shown that polished monolithic zirconia has the lowest wear rate on an enamel antagonist compared to veneered zirconia, glazed zirconia using a glaze spray, monolithic base alloy, or glazed zirconia using glaze ceramic [18, 19]. However, the use of night guard is recommended after the delivery of the final monolithic zirconia to prevent wear of the opposing dentition.

Screw-retained implant restorations are often chosen because they offer better retrievability, decreased space requirements, and healthier soft tissue. On the other hand, cement-retained restorations offer improved occlusal accuracy, enhanced esthetics, increased chances of achieving a passive fit, and decreased instances of retention loss [18]. Moreover, some systematic reviews have shown that differences between cement- and screw-retained restorations are not statistically significant. These reports concluded that screw-retained restorations are equally suitable [18–20]. However, the preferred technique in this study was screw-retained implant restoration due to their retrievability, less biological complications, and easy repair of technical complications. In accordance to this idea, 88% of the restorations in this study were screw-retained. No complications such as screw loosening or decementation were reported in the present study. And only one veneer chipping was found after 1-year follow-up.

## 5. Conclusions

1. Monolithic zirconia CAD/CAM-milled framework restorations are a treatment option for full arch restorations over implants, showing a 96% success rate in the present study. Some of the benefits are accuracy, reduced veneering porcelain, and minimal occlusal adjustments. The outcome of the present study showed high success in function, aesthetics, phonetics, and high patient satisfaction.
2. A full occlusal contour monolithic framework can diminish chipping of the veneered porcelain. However, the fabrication is technique sensitive and should follow the appropriate steps discussed in this study.
3. The digital cut back for veneer placement in the monolithic zirconia was an effective option to avoid surface roughness that can produce crack propagation and veneer chipping.
4. Twenty-three of 26 restorations were screw-retained due to their retrievability, less biological complications, and easy repair of technical complications. Only, one veneer chipping was found in one these restorations. Of the three cement-retained restorations no complications were reported.
5. Within the limitations of the present study monolithic zirconia CAD/CAM milled prosthetic restorations were a successful treatment option for full arch implants supported restorations.
6. More long-term data studies are required for the full arch monolithic zirconia restorations in order to evaluate success and complications over the time.

## Figures and Tables

**Table 1:** Literatures of monolithic zirconia.

Author	Publication date	Study type	*N* (number of arches)
Papaspyridakos and Lal [12]	2008	Case report	1
Lazetera [13]	2009	Case report	1
Papaspyridakos and Lal [9]	2010	Case report	1
Larsson et al. [11]	2010	Prospective study	10
Rojas-Vizcaya [1]	2011	Case report	2
Sadid-Zadeh et al. [5]	2013	Case report	1
Pozzi et al. [10]	2013	Retrospective study	26

**Table 2:** Results of 14 cases in which monolithic zirconia framework for full arch implant supported reconstruction was used.

Subjects	Location	Number of implants	Type of restoration	Time of follow-up	Complications
1	Mandible	4	Screw-retained	10 months	None
	Maxilla	6	Screw-retained	10 months	None

2	Mandible	4	Screw-retained	1 year, 10 months	None
	Maxilla	7	Screw-retained	1 year, 10 months	None

3	Mandible	6	Screw-retained	1 year, 8 months	None
	Maxilla	6	Screw-retained	1 year, 8 months	None

4	Mandible	4	Screw-retained	5 months	None
	Maxilla	6	Screw-retained	5 months	None

5	Mandible	6	Screw-retained	1 year	None
	Maxilla	6	Screw-retained	1 year	None

6	Mandible	4	Screw-retained	7 months	None
	Maxilla	6	Screw-retained	7 months	None

7	Mandible	4	Screw-retained	3 months	None
	Maxilla	4	Screw-retained	3 months	None

8	Mandible	4	Screw-retained	1 year, 3 months	None
	Maxilla	8	Screw-retained	1 year, 3 months	Chipping #9, at 1-year follow-up

9	Mandible	6	Screw-retained	10 months	None
	Maxilla	7	Screw-retained	10 months	None

10	Mandible	6	Screw-retained	2 years	None
	Maxilla	8	Screw-retained	2 years	None

11	Mandible	7	Screw- and cement-retained	3 years, 6 months	None
	Maxilla	8	Screw- and cement-retained	3 years, 6 months	None

12	Maxilla	8	Screw-retained	3 years, 6 months	None

13	Maxilla	9	Screw-retained	1 year	None

14	Maxilla	6	Screw-retained	4 months	None
	Mandible	6	Screw-retained	4 months	None
